# Research Methods in Aging Research

**DOI:** 10.1093/geroni/igaf122.2038

**Published:** 2025-12-31

## Abstract

In this session research designs and methods are described to help inform clinicians and researchers in distinguishing impact.

